# HIV testing and seroprevalence among couples of people diagnosed with HIV in China: A meta-analysis

**DOI:** 10.1371/journal.pone.0247754

**Published:** 2021-03-19

**Authors:** Ci Zhang, Han-Zhu Qian, Xi Chen, Scottie Bussell, Yan Shen, Honghong Wang, Xianhong Li

**Affiliations:** 1 Xiangya School of Nursing, Central South University, Changsha, Hunan Province, China; 2 Xiangya Center for Evidence-Based Nursing Practice & Healthcare Innovation (A JBI Affiliated Group), Changsha, Hunan Province, China; 3 School of Public Health, Yale University, New Haven, Connecticut, United States of America; 4 Hunan Provincial Central for Disease Control and Prevention, Changsha, Hunan Province, China; 5 Department of Health and Human Services, Parker Indian Hospital, Parker, Arizona, United States of America; Indiana University School of Medicine, UNITED STATES

## Abstract

**Background:**

Partner notification and testing could expand HIV testing and link infections to care. We performed a meta-analysis on HIV testing rate and prevalence among couples of people diagnosed with HIV in China.

**Methods:**

Six electronic databases (PubMed, Cochrane Library, Embase, Web of Science, the China National Knowledge Internet, and WanFang) and abstracts of five HIV/sexually transmitted infections conferences were searched up to February 1, 2020. Meta-analysis was conducted using a random-effects model to assess HIV testing rate and prevalence among couples of Chinese people diagnosed with HIV.

**Results:**

Of 3,657 records retrieved, 42 studies were identified. Among them, three studies were conducted among pregnant women and 10 among men who have sex with men. The pooled uptake rate of couples HIV testing among Chinese people diagnosed with HIV was 65% (95% confidence interval, 57% -73%; 23 studies). The pooled HIV prevalence among couples who had an HIV test was 28% [24%-32%] (38 studies). Subgroup analyses showed that the pooled couples HIV testing uptake rates among pregnant women and men who have sex with men were 76% [66%-86%] (3 studies) and 49% [30%-68%] (8 studies), and the pooled HIV prevalence in two populations was 53% [27%-78%] (3 studies) and 14% [10%-17%] (10 studies), respectively.

**Conclusions:**

Nearly two-thirds of couples of people diagnosed with HIV have had an HIV test, of whom 28% were positive. Couples of MSM with a positive HIV diagnosis had a lower testing rate, which indicates more effective strategies need to be carried out to improve couples HIV testing among Chinese MSM.

## Introduction

Historically, intravenous drug use played a major role in HIV transmission in China; however, currently, sexual intercourse is the main mode of HIV transmission [1, 2]. Only 68% of people living with HIV (PLWH) are aware of their positive status [3], which is well below from the 90% awareness target by 2020 as set by the Joint United Nations Programme on HIV and AIDS [4]. This suggests that there is still a significant gap of HIV testing in China.

Couples HIV Testing (CHT) is an approach to have couples tested for HIV and promote HIV testing [5]. This strategy can increase their knowledge of their serostatus and encourage disclosure among people who are in an ongoing sexual relationship [6, 7]. Studies show that CHT is a feasible strategy that can expand HIV testing and further, prevent HIV transmission by increasing condom use among discordant couples [8, 9]. In addition, encouraging CHT among people who have been diagnosed with HIV (PDWH) can identify additional HIV-infected individuals and direct them to early antiretroviral therapy (ART) [10].

The World Health Organization (WHO) released guidelines for the testing and counseling of couples in 2012 and strongly recommended CHT as an essential strategy to promote HIV testing and reach more PLWH [7]. In the 13^th^ Five-Year Plan, which mapped out the tasks to build a healthy China, the Chinese government encouraged CHT among PDWH [11]. We conducted a meta-analysis examining CHT uptake rate and HIV prevalence among Chinese PDWH in order to provide a useful summary of evidence on CHT practice and outcomes.

## Materials and methods

This meta-analysis was reported according to the PRISMA guidelines (S1 Table) [12].

### Inclusion criteria

We defined CHT as 1) HIV testing of sexual partners (whether married or not) reported by participants; and 2) testing recorded by the Chinese Centers for Disease Control (CDC) staff. The target population was defined as Chinese PDWH who had documented records of CHT.

Studies were eligible if they reported data on at least one of the following outcomes: the proportion of CHT among Chinese PDWH, and the HIV prevalence among CHT for Chinese PDWH. CHT uptake rate was calculated using the following formula: (number of PDWH couples who had HIV testing) / (number of PDWH). HIV prevalence among CHT was calculated using the following formula: (number of infected couples)/(number of couples who had HIV testing). Randomized controlled trials (RCTs), quasi-experimental studies, and observational (cross-sectional, cohort, and case-control) studies were eligible for inclusion. For experimental trials and cohort studies, baseline data were used for the meta-analysis. Studies were excluded if they were qualitative, a review, or a duplicate report.

### Search strategy

**Fig 1:**
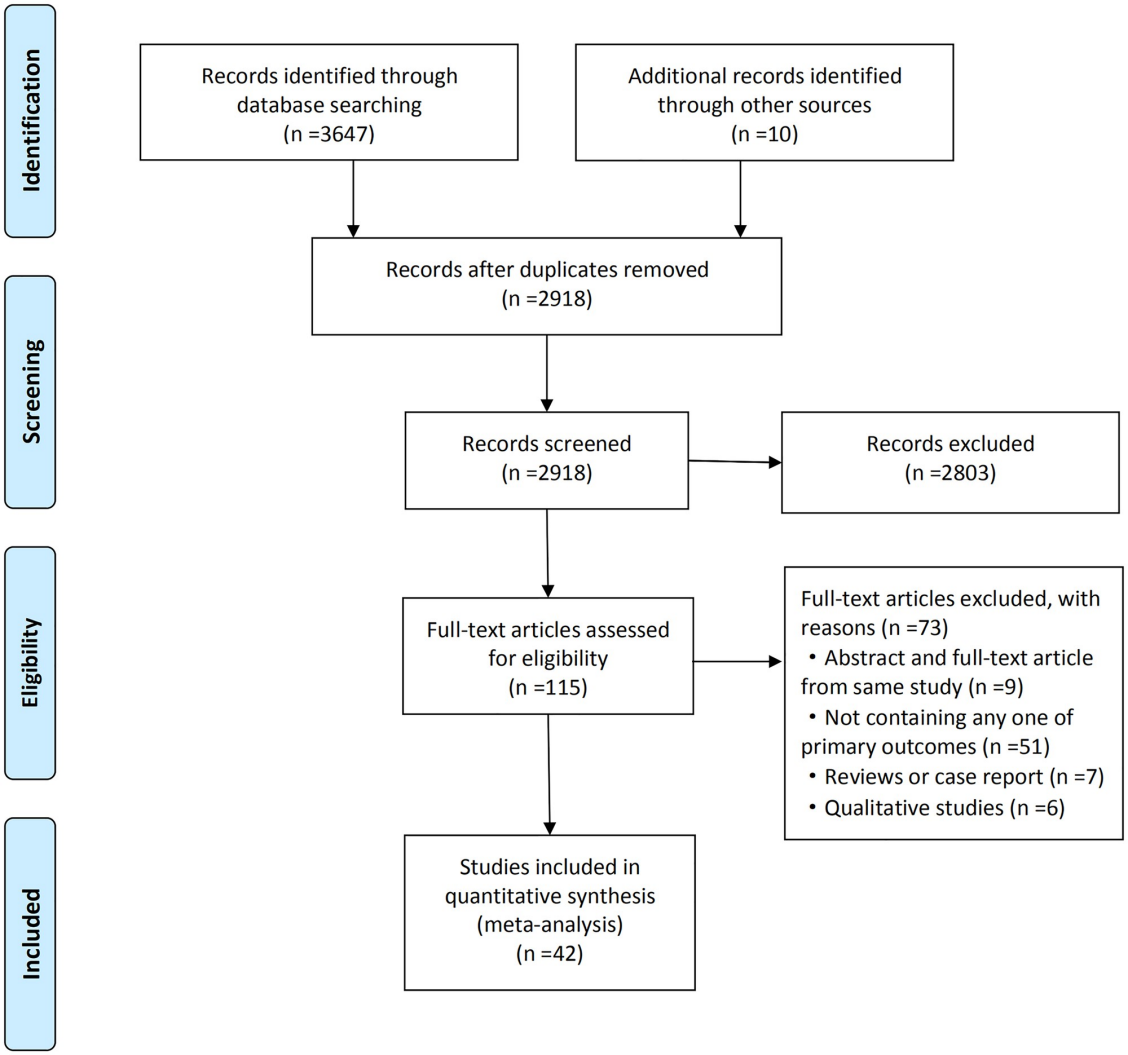
Flow chart of literature search and selection procedures and outcomes.

We searched six electronic databases (PubMed, Embase, Web of Science, Cochrane Library, the China National Knowledge Internet, and WanFang) and abstracts from the International AIDS Society (IAS), HIV Diagnostics Conference (HDC), Canadian Association of HIV Research (CAHR), Infectious Diseases Society of America (IDSA), and the International Congress of Behavioral Medicine (ICBM) for publications up to February 1, 2020. Our search terms included (China OR Chinese) AND ((“couple HIV testing” OR “couples HIV testing” OR “partner HIV testing” OR “partner testing” OR “couple testing” OR “couples testing”) OR ((test OR testing) AND ("couple" OR "couples" OR "partner" OR "partners"))) AND "HIV Infections"[MeSH] OR "HIV infections" OR "HIV infection" OR "Acquired Immunodeficiency Syndrome"[MeSH] OR "Acquired Immunodeficiency Syndrome" OR "Acquired Immunodeficiency Syndromes" OR AIDS OR HIV). The search was limited to human studies, and English and Chinese language publications. We included abstracts if full texts could not be accessed, and we contacted the authors for original data if needed. Gray literature was screened using Google Scholar. In addition, the reference lists of included studies and previously published reviews were searched for additional potentially eligible studies. The literature search and study selection procedures are described in Fig 1.

### Data screening and extraction

Two reviewers (C.Z. and Y.S.) independently screened the titles and abstracts of the articles referring to Chinese PDWH and CHT, and then screened full texts for eligibility. Discrepancies (about 5%) were resolved by discussions with a third reviewer (X.L.). A standard data extraction form was used to extract the variables (including author, year, province, study design, study period, sample size, population, type of union, outcome measurement, CHT uptake, and HIV prevalence among CHT) from the identified studies.

### Quality assessment

We performed a quality assessment of the included studies using the Joanna Briggs Institute Critical Appraisal Checklist for cross-sectional studies [13]. RCTs, quasi-experimental studies, cohort studies, and case-control studies were also evaluated using the cross-sectional study checklist, as data was only extracted from the baseline phase. The checklist has eight items, so the total score for each study ranged from 0 to 8, and it was categorized as low quality ≤ 3, moderate = 4–6, and high quality ≥ 7. The quality assessment was conducted by two independent reviewers (C.Z. and Y.S.), and the disagreements were resolved by discussion with a third reviewer (X.L.).

### Statistical analysis

STATA 12.0 was used to summarize the results. A meta-analysis was conducted using the DerSimonian-Laird random-effect model to produce pooled proportions and 95% confidence intervals (95% CI) [14, 15]. Heterogeneity was assessed using *I*^*2*^ statistics. In addition, subgroup analyses were performed to explore the source of heterogeneity by the study population and the type of couples. In this study, we classified couples into two types: couples that were defined as people in an ongoing sexual relationship and spouses that were defined as people who have a legal marital relationship. Egger’s tests and funnel plot visual inspection were performed to detect publication bias [16]. A sensitivity analysis was performed to detect the impact of each study on the pooled estimate using the leave-one-out approach, which is a repeating procedure of removing one study from the analysis each time. There are two ways to determine that one study impacts the pooled estimate significantly: the estimate after removing this study is across the upper or lower CI limit lines of the pooled estimate; the upper or lower CI limit lines after removing this study are across the pooled estimate line.

## Results

### Description of included studies

A total of 3,657 records were retrieved, of which 3,647 were from database searching and 10 from other sources. After removing duplicates, 2,918 records were identified and 2,803 records were excluded by reviewing the title or abstract. The eligibility of the remaining 115 records was assessed by reviewing the full-text articles, and 73 studies were excluded. Finally, 42 studies were included in the meta-analysis (Fig 1).

These included studies were conducted between 1989 and 2019 and published during 2009–2019, which are presented in Table 1 [17–58]. Thirty-eight studies were published in Chinese [17–51, 54, 55, 58] and four in English [52, 53, 56, 57]. Geographically, the 42 included studies covered 20 provinces (58.8%) of all 34 provinces in China, with 13 studies conducted in Yunnan Province [17, 18, 21–23, 27, 31, 35, 38, 39, 51, 56, 58] and 7 studies in Guangxi Province [19, 34, 36, 41, 42, 44, 51]. The sample size varied from 28 to 48,931. Most studies were cross-sectional (83.3%, 35/42) [17, 19–22, 25–30, 32–45, 47–55, 58]. The majority of studies were conducted among general PDWH (27/42, 64.3%) [18, 20, 22, 26, 27, 29, 32–52, 54], 11 (23.8%) among men who have sex with men (MSM) [19, 23–25, 28, 30, 31, 52, 55–57], 3 (7.1%) among pregnant women [17, 21, 58], and one (2.4%) among blood transfusion recipients [53]. In most studies (85.7%) [19, 20, 22–33, 35–47, 49–56, 58], the outcomes of CHT uptake and HIV infection were recorded by a CDC staff member when PDWH couples underwent HIV testing in a CDC. Reported outcome variables included uptake of CHT (23 studies) [17–21, 24, 25, 27–31, 33, 34, 39, 40, 44, 46–48, 55, 57, 58] and HIV prevalence among CHT (38 studies) [17, 20–43, 45, 47–58]. The quality of each study was evaluated in detail (S2 Table). Most studies were assessed as high quality (38/42, 90.5%) [17–34, 36–38, 41–43, 45–58].

**Table 1:** Study characteristics and outcomes of Chinese couples HIV testing (CHT).

	Study characteristics							Outcomes			Quality score
No.	Publication	Province	Study design	Study period	Sample size*	Population	Type of union	Outcome measurement	CHT uptake (%)**	HIV prevalence among CHT (%)***	
1	Zheng [17], 2019	Yunnan	CS	Jan 2012-Jun 2016	5086	Pregnant women	Couples	Self-report	81.3	32.7	8
2	Yu [18], 2017	Yunnan	CC	Jul 2012-Sep 2015	223	Unspecified	Spouses	Self-report	25.1	NA	8
3	Lan [19], 2017	Guangxi	CS	Until Nov 2016	405	MSM	Spouses	Observation	48.1	NA	8
4	Zhao [20], 2017	Jiangsu	CS	Until Dec 2015	158	Unspecified	Couples	Observation	74.7	18.6	8
5	Wang X [21], 2015	Sichuan, Yunnan, Xinjiang	CS	Jan 2012-Dec 2014	2007	Pregnant women	Spouses	Self-report	69.7	63.6	8
6	Bai [22], 2016	Yunnan	CS	Jan 2014-Dec 2015	263	Unspecified	Spouses	Observation	NA	30.4	8
7	Li Q [23], 2016	Yunnan	QE	May 2014-Dec 2015	105	MSM	Couples	Observation	NA	15.2	8
8	Li [24], 2019	Liaoning	RCT	Aug 2017-Jan 2019	94	MSM	Couples	Observation	17.0	26.3	8
9	Chen [25], 2019	Zhejiang	CS	Sep 2015-Sep 2016	321	MSM	Couples	Observation	41.1	13.8	8
10	Xu [26], 2013	Hebei	CS	Jan 1989-Dec 2011	232	Unspecified	Couples or spouses	Observation	NA	20.7	8
11	Xu [27], 2014	Yunnan	CS	Jan 1995-Dec 2013	2762	Unspecified	Couples or spouses	Observation	88.7	49.0	8
12	Wang [28], 2018	Jiangsu	CS	Jan 2010-Dec 2016	199	MSM	Couples	Observation	80.0	10.5	8
13	Lian [29], 2019	Fujian	CS	Jan 2015-Dec 2018	2937	Unspecified	Spouses	Observation	89.9	20.5	7
14	Da [30], 2019	Hubei	CS	Jan 2013-Dec 2017	2772	MSM	Spouses	Observation	28.9	18.7	8
15	Li Y [31], 2016	Yunnan	QE	May 2014-Dec 2015	118	MSM	Couples	Observation	60.2	13.1	8
16	Liu [32], 2018	Shanxi	CS	Until Nov 2015	246	Unspecified	Couples or spouses	Observation	NA	24.0	8
17	Wang M [33], 2015	Guangdong	CS	Jan 2010-Dec 2012	213	Unspecified	Spouses	Observation	82.2	41.1	8
18	Hu [34], 2014	Guangxi	CS	Aug 2012-Dec 2013	425	Unspecified	Couples or spouses	Self-report	70.4	40.5	8
19	Zhu [35], 2010	Yunnan	CS	Jan 1990-Sep 2009	196	Unspecified	Spouses	Observation	NA	52.0	6
20	Zhong [36], 2016	Guangxi	CS	Jan 2015-Dec 2015	45	Unspecified	Couples or spouses	Observation	NA	26.7	7
21	Chen [37], 2018	Anhui	CS	Jan 2000-Aug 2016	231	Unspecified	Spouses	Observation	NA	20.3	8
22	Duan [38], 2004	Yunnan	CS	March 2003	84	Unspecified	Spouses	Observation	NA	19.0	8
23	Xi [39], 2009	Yunnan	CS	Jan 1996-Dec 2008	88	Unspecified	Couples or spouses	Observation	83.0	31.5	6
24	Xu [40], 2011	Beijing	CS	NA	451	Unspecified	Spouses	Observation	84.7	7.3	6
25	Zhu [41], 2014	Guangxi	CS	NA	409	Unspecified	Spouses	Observation	NA	34.7	8
26	Chen J [42], 2018	Guangxi	CS	Jan 2006-Dec 2015	1658	Unspecified	Spouses	Observation	NA	34.1	8
27	Chen [43], 2015	Fujian	CS	Jan 2008-Dec 2013	872	Unspecified	Couples or spouses	Observation	NA	26.5	8
28	Nong [44], 2019	Guangxi	CS	Before Apr 2014	1307	Unspecified	Couples or spouses	Observation	76.3	NA	6
29	Yang [45], 2018	Jiangxi	CS	Jan 2017-Dec 2017	765	Unspecified	Spouses	Observation	NA	31.5	8
30	Yang [46], 2019	Jiangxi	RCT	Jan 2018-Dec 2017	206	Unspecified	Couples or spouses	Observation	50.0	NA	8
31	Wang [47], 2008	Shandong	CS	Jan 2003-Jun 2007	62	Unspecified	Spouses	Observation	66.1	39.0	8
32	Zhang [48], 2015	Shanghai	CS	Jul 1998-Jul 2014	307	Unspecified	Couples or spouses	Self-report	73.9	32.5	8
33	Zeng [49], 2010	Sichuan	CS	Jan 2008-March 2008	226	Unspecified	Spouses	Observation	NA	25.7	8
34	Zhang [50], 2013	Xinjiang	CS	Aug 2010-Feb 2011	383	Unspecified	Couples or spouses	Observation	NA	39.4	8
35	Li J [51], 2016	Yunnan, Henan, Sichuan, Guangxi, Xinjiang	CS	Jan 2011-Dec 2014	48931	Unspecified	Spouses	Observation	NA	24.6	8
36	Lian [52], 2018	Beijing, Jiangsu, Shanxi, Chongqing, Zhejiang, Hubei	CS	Apr 2014- Dec 2015	829	MSM	Couples	Observation	NA	11.0	8
37	Chen S [53], 2018	Hebei	CS	Jan 1995-Dec 2015	285	Blood transfusion recipients	Spouses	Observation	NA	20.8	7
38	Lin [54], 2010	Zhejiang	CS	May 2008-Mar 2010	129	Unspecified	Couples or spouses	Observation	NA	47.3	7
39	Li J [55], 2017	Unknown	CS	Jan 2014-Jun 2015	5081	MSM	Spouses	Observation	73.1	7.6	7
40	Fu [56], 2016	Zhejiang, Yunnan	QS	June 2014-May 2015	275	MSM	Couples	Observation	NA	10.5	8
41	Mi [57], 2015	Sichuan	QS	Dec 2008-Sep 2009	160	MSM	Couples	Self-report	45.6	25.6	8
42	Qiu [58], 2009	Yunnan	CS	Jul 2005-Jue 2006	28	Pregnant women	Spouses	Observation	78.6	63.6	7

NA, no data available; CS, cross-sectional; RCT, randomized controlled trial; QE, quasi-experimental study; CC, case-control study; PDWH, people diagnosed with HIV; Unspecified means no specific classification on the population of PDWH; MSM, men who have sex with men.

*Sample size was based on the number of PDWH.

** CHT uptake was calculated by the formula: (number of PDWH couples who had HIV testing) / (number of PDWH).

*** HIV prevalence among CHT (%) was calculated by the formula: (number of infected couples)/(number of couples who had HIV testing). Couples were defined as people in an ongoing sexual relationship. Spouses were defined as people who have a legal marital relationship.

### Uptake of CHT

**Fig 2:**
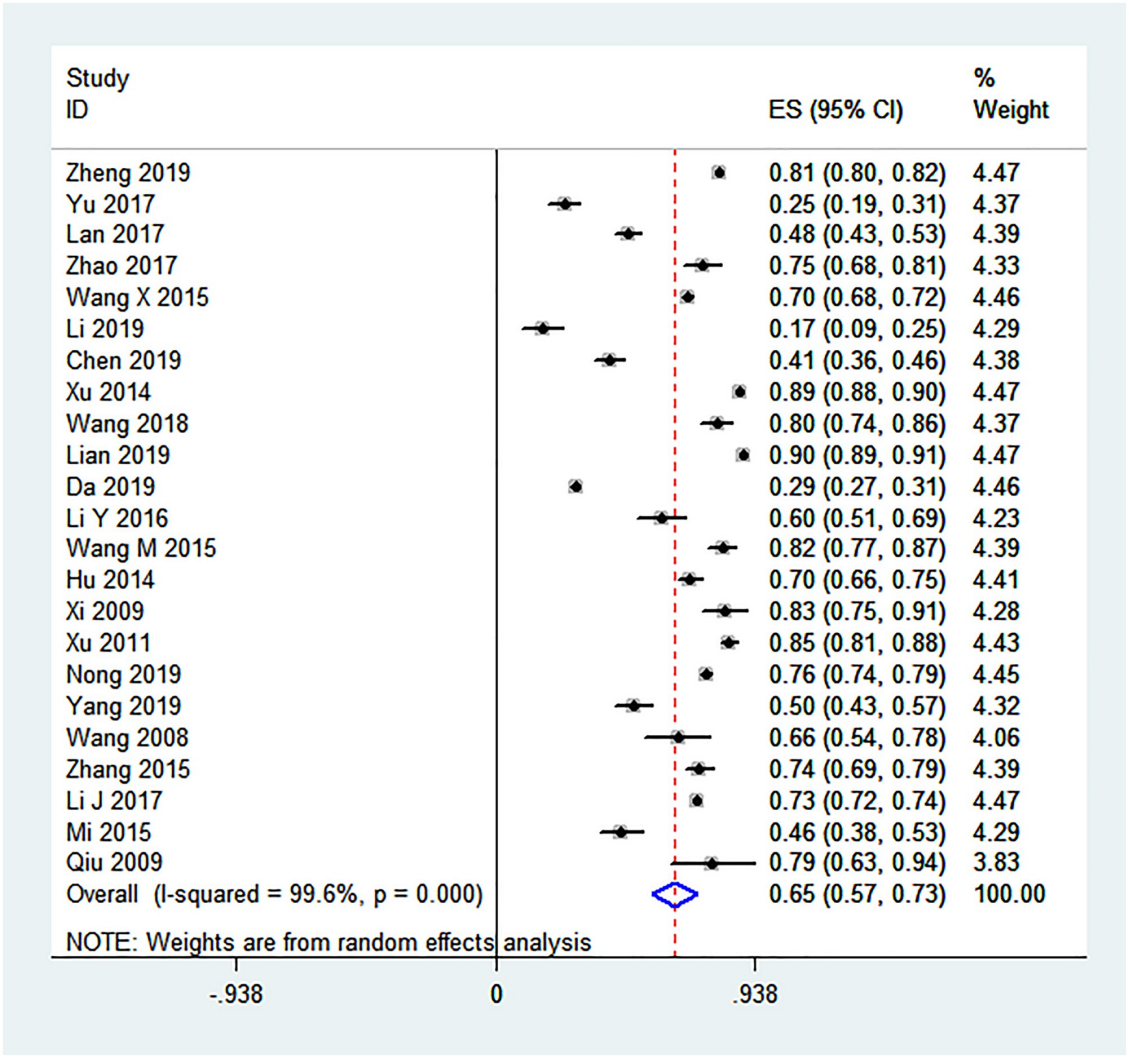
Forest plot of CHT uptake among Chinese PDWH.

The pooled proportion for the uptake of CHT among Chinese PDWH was 65% (95% CI: 57%–73%). Significant heterogeneity was observed between individual studies included in the analysis (*I*^*2*^ = 99.6%, *P* < 0.001) (Fig 2).

### HIV prevalence among PDWH couples

**Fig 3:**
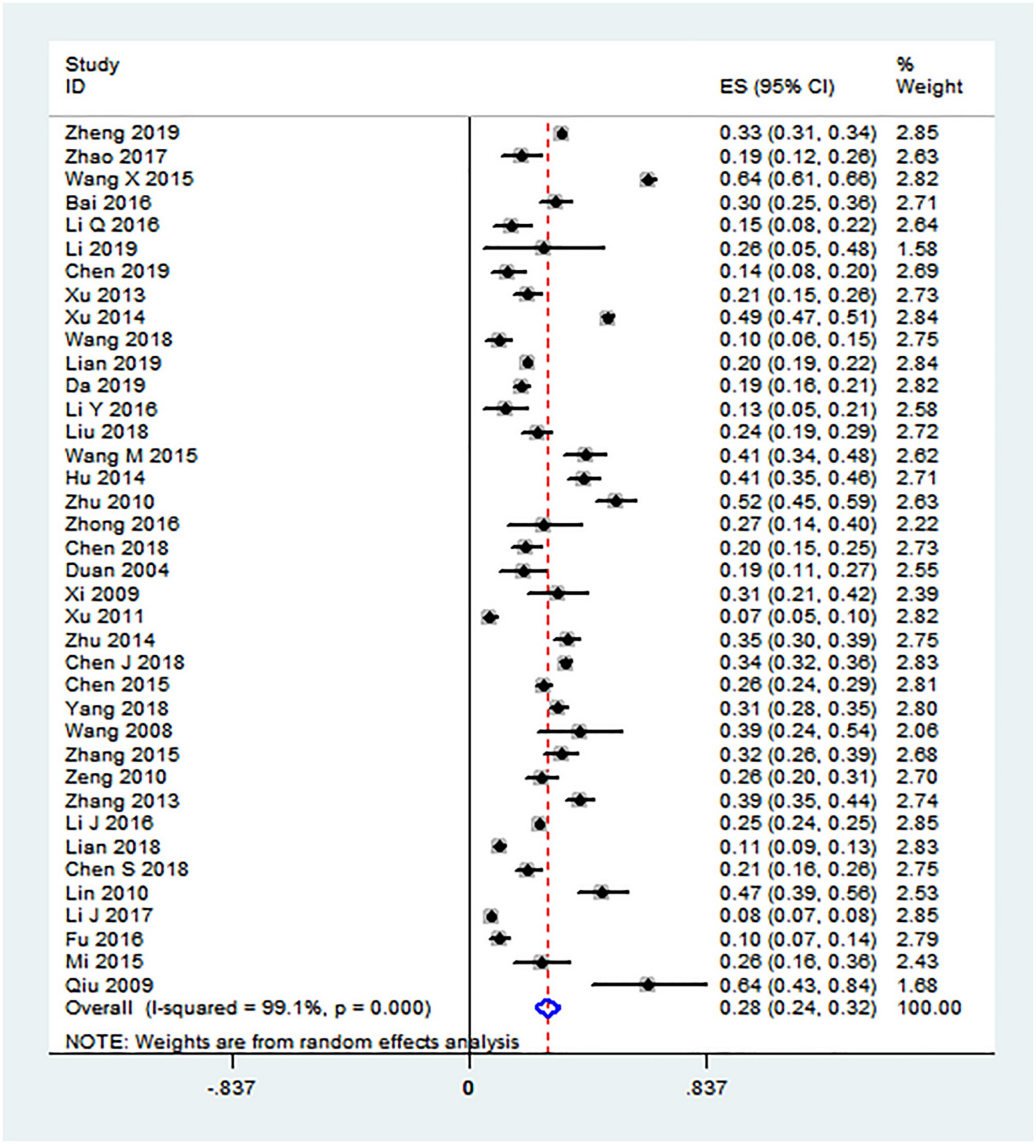
Forest plot of HIV prevalence among couples of Chinese PDWH.

Among Chinese PDWH couples, the pooled HIV prevalence was 28% (95%CI: 24%–32%) (Fig 3). Significant heterogeneity, observed between individual studies, was included in the analysis (*I*^*2*^ = 99.1%, *P* < 0.001).

### Subgroup analysis

The subgroup analyses are shown in Table 2. The pooled uptake rate of CHT among pregnant women (76%, 95%CI: 66%–86%) was higher than that of MSM (49%, 95%CI: 30%–68%). Of the eight studies among MSM participants, three reported that their sexual partners were their spouses (legally married women). The uptake rate of CHT among PDWH couples (49%, 95%CI: 27%–70%) was similar to that among spouses (50%, 95%CI: 17%–83%). The CHT uptake rate in Yunnan province (69%, 95%CI: 59%–80%) was slightly higher than that in Guangxi province (65%, 95%CI: 49%–81%) (Table 2).

**Table 2:** Subgroup analyses of uptake rate of CHT and HIV seroprevalence among PDWH couples.

Characteristic	Study, No.	ES (95% CI)	*I*^*2*^, %	*P* value for heterogeneity
Uptake rate of CHT by study population				
Pregnant women	3	0.76 (0.66–0.86)	98.0	<0.001
MSM	8	0.49 (0.30–0.68)	99.6	<0.001
Uptake rate of CHT by type of partners				
Couples	5	0.50 (0.17–0.83)	98.0	<0.001
Spouses	3	0.49 (0.27–0.70)	99.9	<0.001
Uptake rate of CHT by province*				
Yunnan	6	0.69 (0.59–0.80)	99.0	<0.001
Guangxi	3	0.65 (0.49–0.81)	98.1	<0.001
HIV prevalence in CHT by study population				
Pregnant women	3	0.53 (0.27–0.78)	99.5	<0.001
MSM	10	0.14 (0.10–0.17)	89.2	<0.001
HIV prevalence in CHT by type union among MSM study participants				
Couples	8	0.13 (0.10–0.15)	40.9	0.106
Spouses	2	0.13 (0.02–0.24)	98.3	<0.001
HIV prevalence in CHT by province**				
Yunnan	9	0.33 (0.25–0.42)	97.3	<0.001
Guangxi	4	0.35 (0.32–0.39)	49.2	0.116

CHT, couples’ HIV testing; PDWH, people diagnosed with HIV; MSM, men who have sex with men.

* One study was excluded for analysis [21], which only reported the total uptake rate of CHT from several provinces.

** Three studies were excluded for analysis [21, 35, 40], which only reported total HIV prevalence in CHT from several provinces.

Subgroup analysis of HIV seroprevalence among CHT showed that the pooled prevalence among couples with HIV-infected pregnant women (53%, 95%CI: 27%–78%) was higher than that among couples of MSM (14%,95%CI: 10%–17%). Of the studies among MSM participants, two studies reported that their sexual partners were specifically their spouses. If these two studies were excluded, the pooled HIV prevalence was 13% (95% CI: 2%–24%) with moderate heterogeneity (*I*^*2*^ = 40.7%, *P* = 0.106) (Table 2). The pooled HIV prevalence in Guangxi province (35%, 95%CI: 32%–39%) was slightly higher than that in Yunnan province (33%, 95%CI: 25%–42%), with moderate heterogeneity (*I*^*2*^ = 49.2%, *P* = 0.116).

### Publication bias

**Fig 4:**
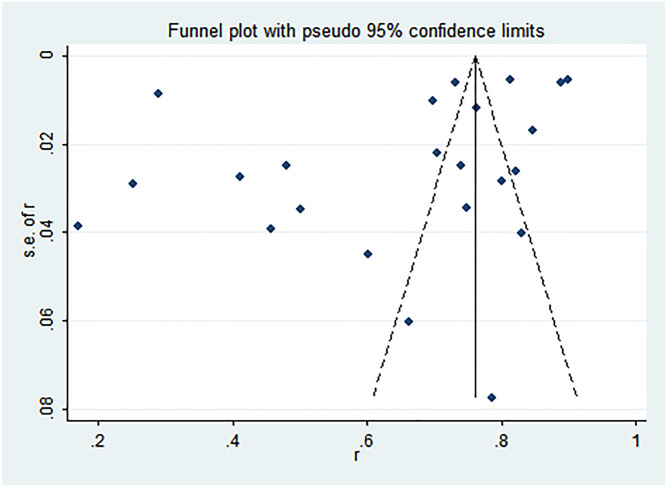
Funnel plot of CHT uptake rate among PDWH couples.

**Fig 5:**
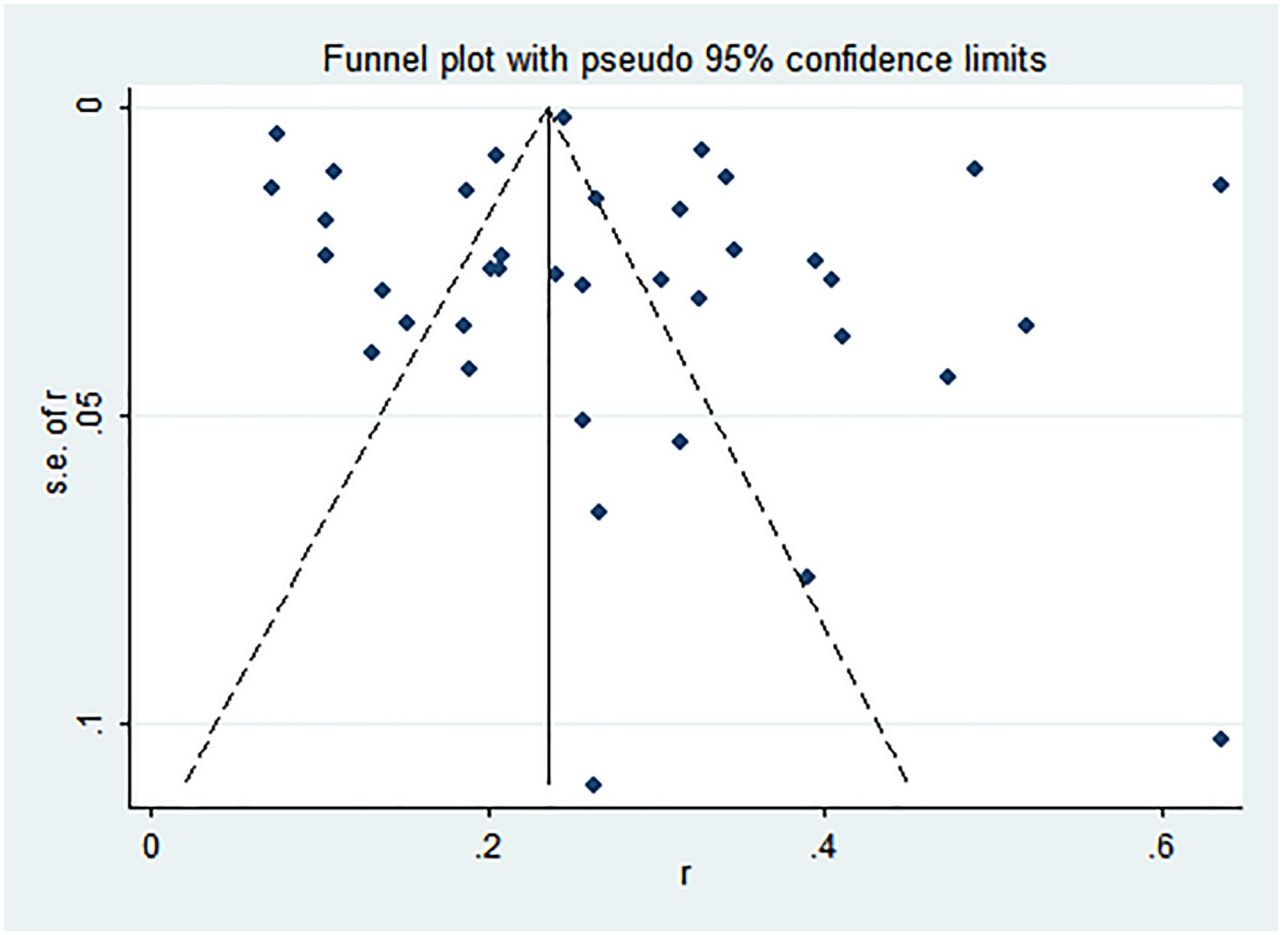
Funnel plot of HIV seroprevalence among PDWH couples.

For the meta-analyses of CHT uptake rate and HIV prevalence among partners of PDWH, both Egger’s (*t* = -1.74, *P* = 0.097; *t* = 0.91, *P* = 0.369) tests found no statistically significant difference, which indicates that there was no publication bias. However, results from the funnel plot of CHT uptake rate among PDWH couples showed that there might be missing studies at the bottom right of the graph (Fig 4), while results from the funnel plot of HIV prevalence among PDWH couples indicated that there might be missing studies from the bottom left of the graph (Fig 5).

### Sensitivity analysis

**Fig 6:**
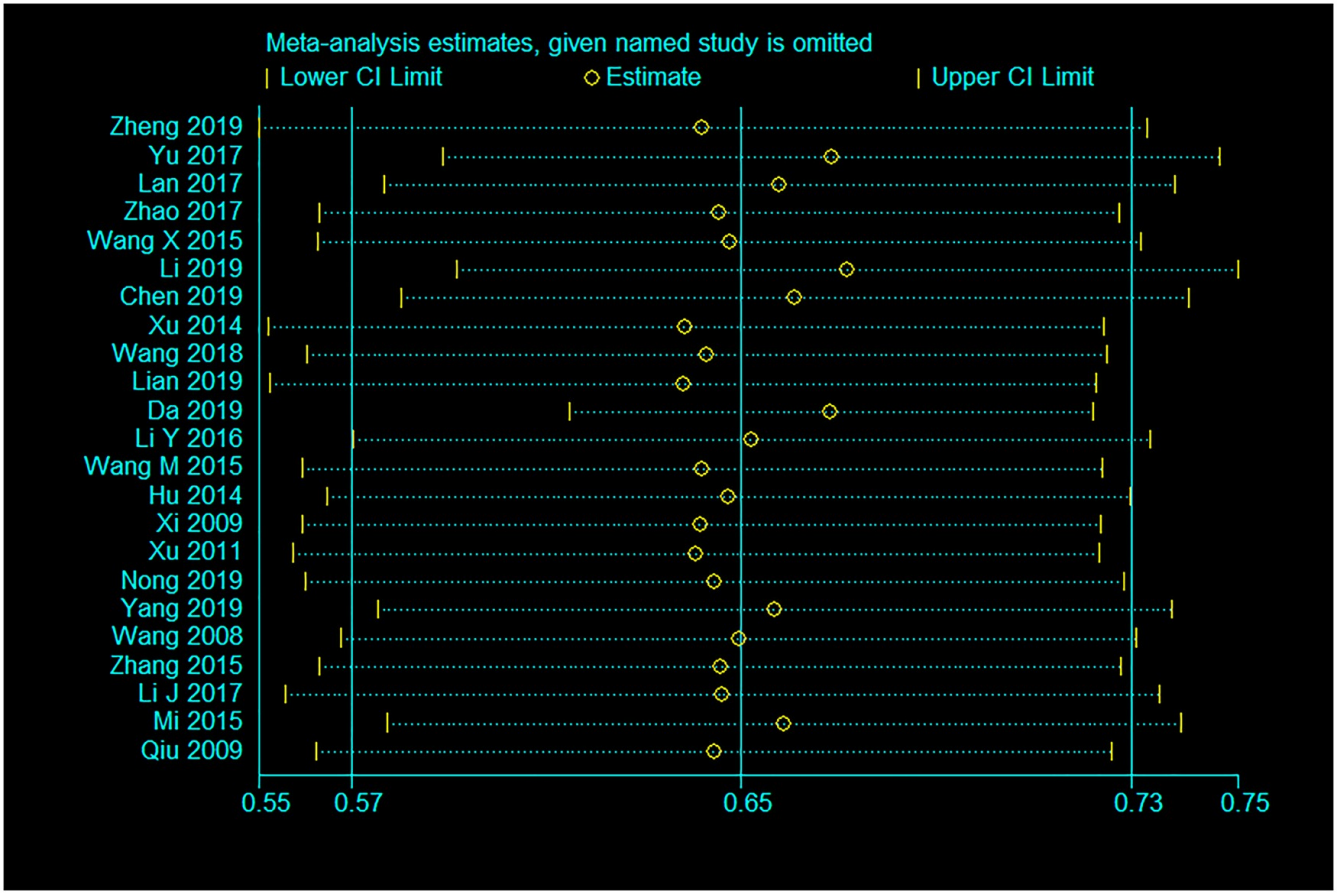
Sensitivity analysis for pooled uptake rate of CHT.

**Fig 7:**
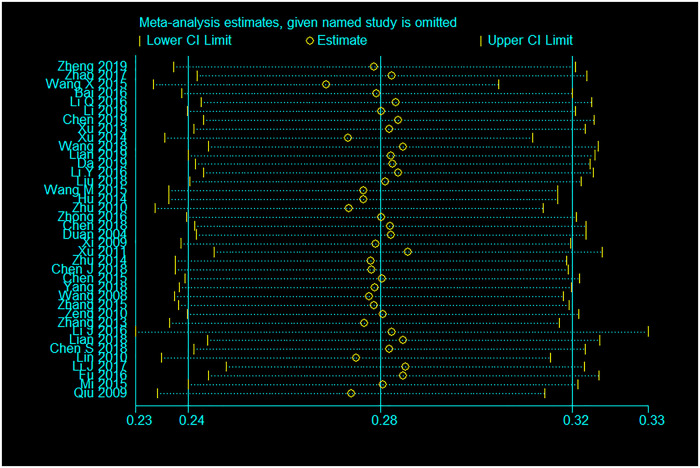
Sensitivity analysis for HIV seroprevalence among PDWH couples.

Sensitivity analyses indicated that none of the included studies significantly changed the pooled estimates for either study outcomes (Figs 6 and 7).

## Discussion

This meta-analysis provided pooled estimates of the uptake rate of CHT and HIV prevalence among Chinese PDWH couples, and presented the data according to study population, type of couple, and province. The CHT uptake rate was 65% among PDWH in China and 49% among couples of HIV-infected MSM. The results suggested that there were gaps in HIV testing among discordant sexual partners. The meta-analysis showed a pooled HIV prevalence of 28% among the PDWH couples in China. Our results highlighted the long-way PDWH couples have to go in order to achieve the Chinese government’s goal to reduce HIV transmission rates between discordant spouses to below 1% by 2030 [11].

In the WHO guidelines for the “Partner Notification Policy,” partner notification relied on the PDWH themselves to notify their sexual partners and receive CHT services [59]. While, taking HIV voluntary counseling and testing is voluntary for PDWH couples across most of China [60], partner notification in the four provinces of Yunnan, Henan, Zhejiang, and Gansu is mandatory among serodiscordant spouses [60]. The WHO guidelines also recommended the promotion of CHT by advocating HIV self-testing (HIVST) among high-risk populations and PDWH couples [59]. Few studies have shown the effectiveness of improved CHT uptake through the distribution of HIVST kits to sexual partners by antenatal and postpartum women [61]. The Chinese government also encouraged the implementation of HIVST and integrated it into routine HIV testing services [11]. However, there is no evidence based on rigorously designed studies that have explored the effects of HIVST on CHT uptake among PDWH in China.

CHT uptake rate is reasonably high among pregnant women study participants (76%), where the purpose may have been the prevention of mother-to-child transmission [62]. The uptake rate was only 49% among HIV-infected MSM study participants. HIV-infected Chinese MSM may have a low rate of disclosure to their sexual partners because of the high levels of stigma and discrimination [59]. In addition, married MSM may have been concerned about the negative consequences of disclosing their sexual orientation to their female spouses [19]. These may have also led to a low CHT uptake. The CHT uptake in Guangxi province (65%) was similar to the pooled CHT uptake rate, but slightly lower than that in Yunnan province (69%). This may be due to the mandatory CHT policy among serodiscordant spouses in Yunnan province [60].

The pooled HIV prevalence in CHT was 28%, which is much higher than that among key populations in China, including MSM, injecting drug users, and sex workers [63, 64]. It is suggested that promoting CHT could be an efficient strategy to identify new infections. HIV prevalence among couples of infected pregnant women (53%) was 3.79 times higher than that among couples of infected MSM (14%). Pregnant women are not typically regarded as a high-risk population and consistent condom use is low with their partners. A recent study showed that 69% of pregnant women and their couples reported inconsistent condom use [65]. HIV prevalence rates among couples and spouses of MSM were similar. The implication may be that the wives of MSM could also be a high-risk population for HIV infection, since up to 70% of Chinese MSM would get married with women under the “filial piety” culture belief [66]. HIV prevalence rates among CHT in Yunnan (33%) and Guangzi (35%) provinces were similar, but much higher than the pooled HIV prevalence (28%). The main reason may be that both provinces have the highest HIV prevalence in China [67].

## Limitations

This meta-analysis had several limitations. First, selection bias could not be ruled out because the languages of the included studies were limited to English and Chinese. Second, information bias was likely to have existed because 14.3% of outcomes were evaluated by the participants’ self-reporting. Third, the heterogeneity across the included studies was high, which may account for publication bias and limit generalizability of the findings. The main reasons might be that samples were recruited from 20 provinces with diverse HIV prevalence and partner notification policies. In addition, some studies had small sample sizes, which might also contribute to the heterogeneity.

## Conclusions

Two-thirds of Chinese couples living with HIV have had an HIV test, of which 28% were positive. Couples of MSM had a lower HIV testing rate, which indicates that more effective strategies need to be carried out to improve couples’ HIV testing among the Chinese MSM population.

## Supporting information

S1 Table PRISMA 2009 checklist. (DOC)

S2 Table Assessment of methodological quality of cross-sectional studies. (DOC)
