# Supplementary material for: HIV testing and seroprevalence among couples of people diagnosed with HIV in China: A meta-analysis
Source: PLoS One. 2021 Mar 19;16(3):e0247754. doi: 10.1371/journal.pone.0247754 (PMC7978381; doi:10.1371/journal.pone.0247754)
Supplement: S2 Table — (DOC) [file pone.0247754.s002.doc]

**S2 Table. Assessment of methodological quality of cross-sectional studies(n=42)**

| Authors year | Q1 | Q2 | Q3 | Q4 | Q5 | Q6 | Q7 | Q8 | Score |
| --- | --- | --- | --- | --- | --- | --- | --- | --- | --- |
| Zheng 2019 | Y | Y | Y | Y | Y | Y | Y | Y | 8 |
| Yu 2017 | Y | Y | Y | Y | Y | Y | Y | Y | 8 |
| Lan 2017 | Y | Y | Y | Y | Y | Y | Y | Y | 8 |
| Zhao 2017 | Y | Y | Y | Y | Y | Y | Y | Y | 8 |
| Wang X 2015 | Y | Y | Y | Y | Y | Y | Y | Y | 8 |
| Bai 2016 | Y | Y | Y | Y | Y | Y | Y | Y | 8 |
| Li Q 2016 | Y | Y | Y | Y | Y | Y | Y | Y | 8 |
| Li 2019 | Y | Y | Y | Y | Y | Y | Y | Y | 8 |
| Chen 2019 | Y | Y | Y | Y | Y | Y | Y | Y | 8 |
| Xu 2013 | Y | Y | Y | Y | Y | Y | Y | Y | 8 |
| Xu 2014 | Y | Y | Y | Y | Y | Y | Y | Y | 8 |
| Wang 2018 | Y | Y | Y | Y | Y | Y | Y | Y | 8 |
| Lian 2019 | Y | Y | Y | Y | Y | N | Y | Y | 7 |
| Da 2019 | Y | Y | Y | Y | Y | Y | Y | Y | 8 |
| Li Y 2016 | Y | Y | Y | Y | Y | Y | Y | Y | 8 |
| Liu 2018 | Y | Y | Y | Y | Y | Y | Y | Y | 8 |
| Wang M 2015 | Y | Y | Y | Y | Y | Y | Y | Y | 8 |
| Hu 2014 | Y | Y | Y | Y | Y | Y | Y | Y | 8 |
| Zhu 2010 | Y | Y | Y | Y | N | N | Y | Y | 6 |
| Zhong 2016 | Y | Y | Y | Y | Y | N | Y | Y | 7 |
| Chen 2018 | Y | Y | Y | Y | Y | Y | Y | Y | 8 |
| Duan 2004 | Y | Y | Y | Y | Y | Y | Y | Y | 8 |
| Xi 2009 | Y | Y | Y | Y | Y | N | Y | Y | 6 |
| Xu 2011 | Y | Y | Y | Y | Y | N | Y | Y | 6 |
| Zhu 2014 | Y | Y | Y | Y | Y | Y | Y | Y | 8 |
| Chen J 2018 | Y | Y | Y | Y | Y | Y | Y | Y | 8 |
| Chen 2015 | Y | Y | Y | Y | Y | Y | Y | Y | 8 |
| Nong 2019 | Y | Y | Y | Y | N | N | Y | Y | 6 |
| Yang 2018 | Y | Y | Y | Y | Y | Y | Y | Y | 8 |
| Yang 2019 | Y | Y | Y | Y | Y | Y | Y | Y | 8 |
| Wang 2008 | Y | Y | Y | Y | Y | N | Y | Y | 8 |
| Zhang 2015 | Y | Y | Y | Y | Y | Y | Y | Y | 8 |
| Zeng 2010 | Y | Y | Y | Y | Y | Y | Y | Y | 8 |
| Zhang 2013 | Y | Y | Y | Y | Y | Y | Y | Y | 8 |
| Li J 2016 | Y | Y | Y | Y | Y | Y | Y | Y | 8 |
| Lian 2018 | Y | Y | Y | Y | Y | Y | Y | Y | 8 |
| Chen S 2018 | Y | Y | Y | Y | Y | N | Y | Y | 7 |
| Lin 2010 | Y | Y | Y | Y | Y | N | Y | Y | 7 |
| Li J 2017 | Y | N | Y | Y | Y | Y | Y | Y | 7 |
| Fu 2016 | Y | Y | Y | Y | Y | Y | Y | Y | 8 |
| Mi 2015 | Y | Y | Y | Y | Y | Y | Y | Y | 8 |
| Qiu 2009 | Y | Y | Y | Y | Y | N | Y | Y | 7 |

Q= Question; Y=Yes; N= No

Q1: Were the criteria for inclusion in the sample clearly defined?

Q2: Were the study subjects and the setting described in detail?

Q3: Was the exposure measured in a valid and reliable way?

Q4: Were objective, standard criteria used for measurement of the condition?

Q5: Were confounding factors identified?

Q6: Were strategies to deal with confounding factors stated?

Q7: Were the outcomes measured in a valid and reliable way?

Q8: Was appropriate statistical analysis used?
