# Supplementary material for: HIV testing and seroprevalence among couples of people diagnosed with HIV in China: A meta-analysis
Source: PLoS One. 2021 Mar 19;16(3):e0247754. doi: 10.1371/journal.pone.0247754 (PMC7978381; doi:10.1371/journal.pone.0247754)
Supplement: Attachment — Submitted filename: Response to reviewers.docx [file pone.0247754.s003.docx]

Nov 30^th^, 2020

Dr. Qigui Yu, M.D./Ph.D
Academic Editor, PLOS ONE

**Re: Responses to the reviewers’ comments on manuscript ID PONE-D-20-11075 entitled “****HIV testing and seroprevalence among couples of people living with HIV in China: a meta-analysis"**

Dear Dr. Yu,

We really appreciate the reviewers’ and your valuable comments on our manuscript. We have addressed these comments one by one as follows. We have also made track changes in the revised manuscript.

| **Response to the Editor:** | |
| --- | --- |
| **Comments** | **Answers** |
| 1. Please ensure that your manuscript meets PLOS ONE's style requirements, including those for file naming. The PLOS ONE style templates can be found at   https://journals.plos.org/plosone/s/file?id=wjVg/PLOSOne_formatting_sample_main_body.pdf and  https://journals.plos.org/plosone/s/file?id=ba62/PLOSOne_formatting_sample_title_authors_affiliations.pdf | This has been revised according to the PLOS ONE’s style requirements. |
| 2. We suggest you thoroughly copyedit your manuscript for language usage, spelling, and grammar. If you do not know anyone who can help you do this, you may wish to consider employing a professional scientific editing service.   Upon resubmission, please provide the following:   1) The name of the colleague or the details of the professional service that edited your manuscript 2) A copy of your manuscript showing your changes by either highlighting them or using track changes (uploaded as a *supporting information* file) 3) A clean copy of the edited manuscript (uploaded as the new *manuscript* file) | Thanks for your suggestions. We have invited the recommended professional organization (Editage, a division of Cactus Communications) to edit language usage, spelling, and grammar.  The manuscripts of both clean and track change versions were uploaded as suggested. |
| Response to Reviewer #1: | |
| **Comments** | **Answers** |
| 1. The paper is well written and does a great job reporting all necessary components of a systematic review and meta-analysis. The research questions and purpose are clear and straightforward. The search strategy and review methodology were clearly described and appropriate, however the manuscript needs to be reviewed for grammar and syntax, preferably by a native English speaker to improve clarity of your prose. | Thanks for your suggestions. We have invited the professional organization (Editage, a division of Cactus Communications) to edit language usage, spelling, and grammar. |
| 2. First reading: To examine CHT and HIV prevalence among Chinese living with HIV - isn't this 100% prevalence? To be accurate should this be HIV prevalence among Chinese people. Or are you hoping to estimate prevalence of HIV among Chinese people who do not know their status? Or maybe discordant couples?   From further reading, I am assuming Chinese PLWH = Serodiscordant Chinese couples -> This needs to be better clarified and I have pointed it out in a few places, but this needs to be used consistently throughout the paper | Our meta-analysis has two main outcomes. One is the uptake rate of HIV testing among the **couples** of people diagnosed with HIV (PDWH) in China. The other is the HIV seroprevalence among PDWH **couples** who took HIV testing, not the HIV seroprevalence among Chinese people living with HIV.  We have clarified it in the inclusion criteria “*Studies were eligible if they reported data on at least one of the following outcomes: the proportion of CHT among Chinese PDWH, and the HIV prevalence among CHT for Chinese PDWH*” on Page 5, lines 74-76. We have checked and revised it throughout the manuscript. Thanks for your advice. |
| 3. Abstract:   It would be helpful to know how many studies attributed to each of the analysis with the estimates, it would clarify why MSM is stated to have 10 studies, however table 2 shows 8 studies for Uptake rate and 10 for HIV prevalence. | We have added the number of studies: “*The pooled uptake rate of couples HIV testing among Chinese people diagnosed with HIV was 65% (95% confidence interval, 57% -73%; 23 studies). The pooled HIV prevalence among couples who had an HIV test was 28% [24%-32%] (38 studies). Subgroup analyses showed that the pooled couples HIV testing uptake rates among pregnant women and men who have sex with men were 76% [66%-86%] (3 studies) and 49% [30%-68%] (8 studies), and the pooled HIV prevalence in two populations was 53% [27%-78%] (3 studies) and 14% [10%-17%] (10 studies), respectively*” at Page 2, lines 30-37. |
| 4. Line 21-22: "…among couples of Chinese people living with HIV…" - it is unclear whether or not both have HIV (seroconcordant) or only one person has HIV (serodiscordant). Assuming it is the latter, this needs to be written to be explicitly and clear throughout the paper. | We changed it to “couples of people diagnosed with HIV (PDWH)”. The HIV status of PDWH couples was unknown. And original studies reported HIV testing among PDWH couples. Our meta-analysis was to synthesize the uptake rate of HIV testing and seroprevalence among couples. |
| 5. Line 28-29: "We conducted three studies among…" - this is confusing, are there words missing? Should it be, "We conducted a subgroup analysis…"? | Thanks for your advice. We have clarified this as “*Among them, three studies were conducted among pregnant women and 10 among* *men who have sex with men*” at Page 2, lines 28-30. |
| 6. Line 36-37: "Almost two-thirds of Chinese couples living with HIV have had an HIV test, of which 28% were positive" - this implies that of the two-thirds that’s were tested, 28% were HIV positive. Were the 28% HIV-positive among the 65% who were tested or are these estimates separately calculated? | “*HIV prevalence among CHT was calculated using the following formula: (number of infected couples)/(number of couples who had HIV testing)*”. This has been clarified at Page 5, lines 78-79. |
| 7. Introduction:   Line 45-46: the goals of 90-90-90 are: 90% of all people living with HIV will know their HIV status-90% of all people with diagnosed HIV infection will receive sustained antiretroviral therapy-90% of all people receiving antiretroviral therapy will have viral suppression. Testing coverage is not specified in the goals. | We agree. As HIV testing is the key step to know one’s serostatus, so we have added the last sentence in the first paragraph “*This suggests that there is still a significant gap of HIV testing in China*”. |
| 8. Line 53-54: "…encouraging CHT among PLWH could identify additional HIV infected individuals…" This is a very confusing statement, I think PLWH is meant to be "undiagnosed" or "serodiscordant"? Similar language is used throughout the introduction and needs to be clarified. | We have revised the sentence as "*…encouraging CHT among people who have been diagnosed with HIV (PDWH) can identify additional HIV-infected individuals…*” at Page 4, lines 56-57. We have changed it throughout the manuscript. |
| 9. Methods:   Line 70: "…the proportion of CHT among Chinese PLWH…" for clarification, what is the denominator in this? Is it Chinese people living with HIV or Chinese couples or serodiscordant couples? This is somewhat described in the footnote in table 1, but this should be included in the text | Thanks for your suggestions. We have added as the definition for the proportion in the text“*CHT uptake rate was calculated using the following formula: (number of PDWH couples who had HIV testing) / (number of PDWH). HIV prevalence among CHT was calculated using the following formula: (number of infected couples)/(number of couples who had HIV testing).*” at Page 5, lines 76-79. |
| 10. Line 101: what were some of the variables extracted from the included studies? Was timeframe of testing abstracted, lifetime vs recent testing? This could have implications for interpreting results. | We have added this information: “*A standard data extraction form was used to extract the variables (including author, year, province, study design, study period, sample size, population, type of union, outcome measurement, CHT uptake, and HIV prevalence among CHT) from the identified studies*” at Pages 6-7, lines 109-112. |
| 11. Line 118-119: were the couples assumed to be sexually monogamous couples or was this information collected? | No, not necessarily. Some might be while some might not. We reported couple HIV testing based on reporting in the included papers. Also see our response to comment #4 above. |
| 12. Line108: Can you describe what some of these 8 items are assessing? Even in the supporting documents the scoring sheet does not describe the questions. | We have added 8 assessments items in S2 Table: “*Q1: Were the criteria for inclusion in the sample clearly defined? Q2: Were the study subjects and the setting described in detail? Q3: Was the exposure measured in a valid and reliable way? Q4: Were objective, standard criteria used for measurement of the condition? Q5: Were confounding factors identified? Q6: Were strategies to deal with confounding factors stated? Q7: Were the outcomes measured in a valid and reliable way? Q8: Was appropriate statistical analysis used?*” |
| 13. Line 121: What is the threshold for determining if a study does impact the pooled estimate in a meaningful way | A sensitivity analysis was performed to detect the impact of each study on the pooled estimate using the leave-one-out approach which is a repeating procedure of removing one study from the analysis each time. The sensitivity analysis results were presented in Figures 6 and 7.  “*There are two ways to determine that one study impacts the pooled estimate significantly: the estimate after removing this study is across the upper or lower CI limit lines of the pooled estimate; the upper or lower CI limit lines after removing this study are across the pooled estimate line*” (Page 8, Lines 135-139). |
| 14. Results:   The Uptake of CHT and Proportion of HIV among PLWH couples could be presented in tabular format, maybe integrated with table 2? The figures themselves can be difficult to interpret for readers who are not familiar with forest plots. | Thanks for the comment. We think Forest Plot is widely used to present meta-analysis results, so we would like to keep the forest plot, but we elaborated and interpreted the results in texts for readers who are not familiar with these figures. |
| 15. Line 136: Can an additional analysis of CHT and HIV prevalence be conducted among the some of the larger provinces that had a lot of data reported for them? This would be interesting to see. | Thanks for your advice. We have added subgroup analysis by province (in two provinces Yunnan and Guangxi with a large number of studies), and have added in the results: “*The CHT uptake rate in Yunnan province (69%, 95%CI: 59%–80%) was slightly higher than that in Guangxi province (65%, 95%CI: 49%–81%) (Table 2)*” (Page 13, lines 203-205) and “*The pooled HIV prevalence in Guangxi province (35%, 95%CI: 32%–39%) was slightly higher than that in Yunnan province (33%, 95%CI: 25%–42%), with moderate heterogeneity (I2=49.2%, P=0.116)*” (Page 14, lines 212-214).  We have also added the interpretations of the results: “*The CHT uptake in Guangxi province (65%) was similar to the pooled CHT uptake rate, but slightly lower than that in Yunnan province (69%). This may be due to the mandatory CHT policy among serodiscordant spouses in Yunnan province [60]*” (Page 17, lines 272-275) and “ *HIV prevalence rates among CHT in Yunnan (33%) and Guangzi (35%) provinces were similar, but much higher than the pooled HIV prevalence (28%). The main reason may be that both provinces have the highest HIV prevalence in China [67]”* (Page 18, Lines 287-290). |
| 16. Line 178: did any of the subgroup analyses uncover any sources of heterogeneity? | For HIV seroprevalence, there are moderate heterogeneity by excluding two studies (*I^2^*=40.7%*, P*=0.106); there was also moderate heterogeneity by province (*I^2^*=49.2%, *P*=0.116) (see the second paragraph in the section of “Subgroup Analysis”) |
| 17. Line 200: The Eggers test may not have shown statistical evidence of publication bias, however I must disagree about the assessment of the funnel plots. Figure 4 seems to indicate there may be missing studies at the bottom right of the graph. Figure 5 seems to indicate some missing studies from the bottom left of the graph. However, this may not be helpful as these tests and bias assessments do not work well when heterogeneity is high. I think justifying lack of evidence for bias could be from your comprehensive search (of published and gray literature) would better serve the paper rather than the statistical testing. | We agree. We have added: “*However, results from the funnel plot of CHT uptake rate among PDWH couples showed that there might be missing studies at the bottom right of the graph (Fig 4), while results from the funnel plot of HIV prevalence among PDWH couples indicated that there might be missing studies from the bottom left of the graph (Fig 5)*” at Page 15, lines 228-232.  In addition, we have also discussed potential reasons for publication bias: “*Third, the heterogeneity across the included studies was high, which may account for publication bias and limit generalizability of the findings. The main reasons might be that samples were recruited from 20 provinces with diverse HIV prevalence and partner notification policies. In addition, some studies had small sample sizes, which might also contribute to the heterogeneity*” at Page 18, lines 296-301. |
| 18. Discussion:   The couples are now described as discordant sexual partners - This is the language that should be used throughout the paper rather than referring to them as Chinese PLWH couples. | The couple’s HIV status could be negative or unknow. We have revised it as “Chinese PDWH couples" throughout the manuscript (please also see our response to comment #4 above). |
| 19. Are there any efforts to increase serodiscordant couple HIV testing for the Chinese population? Are there any outside China that could be adapted and used? | Yes, national HIV prevention guideline recommended couple HIV testing. We have added:“*While, taking HIV voluntary counseling and testing is voluntary for PDWH couples across most of China [60], partner notification in the four provinces of Yunnan, Henan, Zhejiang, and Gansu is mandatory among serodiscordant spouses [60]. The WHO guidelines also recommended the promotion of CHT by advocating HIV self-testing (HIVST) among high-risk populations and PDWH couples [59]. Few studies have shown the effectiveness of improved CHT uptake through the distribution of HIVST kits to sexual partners by antenatal and postpartum women [61]. The Chinese government also encouraged the implementation of HIVST and integrated it into routine HIV testing services [11]*” （Page 16, Lines 254-262）. |
| 20. Another potential limitation is the generalizability of the findings, meaning how well are these Chinese discordant couples included in the studies representative of all Chinese serodiscordant couples? | In Discussion section, we have added: “*Third, the heterogeneity across the included studies was high, which may account for publication bias and limit generalizability of the findings. The main reasons might be that samples were recruited from 20 provinces with diverse HIV prevalence and partner notification policies. In addition, some studies had small sample sizes, which might also contribute to the heterogeneity*” at Page 18, Lines 296-301. |
| 21. Tables and figures:   The figures need to be labeled and annotated in order for readers to be able to understand the them as a standalone result. | Thanks. The figures have been labeled and annotated. |
| Response to Reviewer #2: | |
| 1. Please review and edit the article to be clearer. While the paper did meet expectations laid out in the abstract and the conclusions were well-thought out and appropriate given the data, there were several areas where more concise/clear language could have been used. | Thanks for your suggestions. We have invited the professional organization (Editage, a division of Cactus Communications) to edit language usage, spelling, and grammar. |

Xianhong Li, PhD
